# Supplementary material for: Design Characteristics and Recruitment Rates for Randomized Trials of Peri-Prosthetic Joint Infection Management: A Systematic Review
Source: Antibiotics (Basel). 2023 Sep 27;12(10):1486. doi: 10.3390/antibiotics12101486 (PMC10604750; doi:10.3390/antibiotics12101486)

Documentation of literature searching provided in support of the systematic review.

Title: Systematic review of patients with proven or probable periprosthetic joint infection who are enrolled in RCTs for diagnosis or treatment

Searching was done with mutual peer review from Cheryl Hamill and Bethwyn Allen.

**Database and platform**	**Database coverage**	**Date of final search**
Medline All (Ovid)	1946 to 25 May 2023	29 May 2023
Embase (Ovid)	1974 to 2023 25 May	29 May 2023
Web Of Science	1997-present	29 May 2023
Cochrane Library		7 June 2023
PubMed		29 May 2023
**STUDY Registries**		
ClinicalTrials.gov		31 May 2023
EU Clinical Trials Register		31 May 2023
WHO International Clinical Trials Registry Platform		31 May 2023
Australian and New Zealand Clinical Trials Registry		31 May 2023

References from all sources searched were exported to an EndNote Library.

NOTE- Embase and OVID are limited to the English language

***Ovid Medline* - Total number of references found - 936**

Filter used for RCTs: - Cochrane Highly Sensitive Search Strategy for identifying randomized trials in MEDLINE: sensitivity- and precision-maximizing version Ovid format (2008 revision) [4.S1 Technical Supplement to Chapter 4: Searching for and selecting studies | Cochrane Training] (Version 6.2, 2021);

- Phase 3 amendment based on *Cooper, C., Varley-Campbell, J., & Carter, P. (2019). Established search filters may miss studies when identifying randomized controlled trials. J Clin Epidemiol, 112, 12-19. doi:10.1016/j.jclinepi.2019.04.002 [39]*

Database(s): **Ovid MEDLINE(R) ALL** 1946 to 25 May 2023- searched 29 May 2023
Search Strategy:

#	Searches	Results
1	surgical wound infection/ or staphylococcal infections/	101757
2	exp arthroplasty, replacement/ or exp joint prosthesis/	95675
3	1 and 2	2819
4	Prosthesis-Related Infections/co, di, dg, mi, su, th [Complications, Diagnosis, Diagnostic Imaging, Microbiology, Surgery, Therapy]	10319
5	bone diseases, infectious/ and (anti-bacterial agents/ or reoperation/ or surgical wound infection/)	199
6	(((joint* or implant* or metalware or metal-ware or prosthe* or periprosthe* or peri-prosthe* or TKA or THA or TJA or TAA) adj5 infection*) or PJI or PJIs or DAIR or (prosthetic adj joint adj infection*)).ti,ab,kw.	23752
7	3 or 4 or 5 or 6	30827
8	(randomized controlled trial or controlled clinical trial).pt. or randomized.ab. or randomised.ab. or placebo.ab. or clinical trials as topic.sh. or randomly.ab. or trial.ab.	1754932
9	phase 3 clinical trial/ or ("phase 3" or phase3 or "phase III" or P3 or "PIII").ti,ab.	82518
10	8 or 9	1795851
11	(hip or hips or knee or knees or shoulder* or elbow* or bone* or joint* or orthopaedic or orthopedic or PJI or PJIs or DAIR or (prosthetic adj joint adj infection*)).ti,ab,kw.	1493597
12	7 and 10 and 11	1072
13	exp animals/ not humans.sh.	5124303
14	12 not 13	1021
15	limit 14 to english language	936

PubMed search to identify unindexed citations (ie, not in Medline) - Total number of references found - 181

Searched - 29 May 2023

((periprosthet* OR prosthet* OR joint implant OR arthroplasty OR metalware) AND (orthopedic OR orthopaedic OR hip OR knee OR shoulder OR elbow) AND (infection OR infected OR infections))

AND (rct* OR random* OR controlled trial) AND (inprocess[sb] OR publisher[sb] OR pubmednotmedline[sb]) (181)

***Ovid Embase* - Total number of references found - 1245**

Phase 3 amendment based on *Cooper, C., Varley-Campbell, J., & Carter, P. (2019). Established search filters may miss studies when identifying randomized controlled trials. J Clin Epidemiol, 112, 12-19. doi:10.1016/j.jclinepi.2019.04.002* [39]

Embase 1974 to 2023 May 25 – Searched 29 May 2023.
Search Strategy:

#	Searches	Results
1	periprosthetic joint infection/	5406
2	*prosthesis infection/ or *staphylococcus infection/ or *bone infection/ or *surgical infection/ or orthopedic surgery/ec	36117
3	exp arthroplasty/ or joint surgery/ or orthopedic surgery/ or exp joint prosthesis/	192411
4	2 and 3	3180
5	(((joint* or implant* or metalware or metal-ware or prosthe* or periprosthe* or peri-prosthe* or TKA or THA or TJA or TAA) adj5 infection*) or PJI or PJIs or DAIR or (prosthetic adj joint adj infection*)).ti,ab,kw.	30414
6	1 or 4 or 5	32991
7	crossover-procedure/ or double-blind procedure/ or randomized controlled trial/ or single-blind procedure/ or (random* or factorial* or crossover* or cross over* or placebo* or (doubl* adj blind*) or (singl* adj blind*) or assign* or allocat* or volunteer*).tw.	2908178
8	phase 3 clinical trial/ or ("phase 3" or phase3 or "phase III" or P3 or "PIII").ti,ab.	171877
9	7 or 8	2989946
10	(hip or hips or knee or knees or shoulder* or elbow* or bone* or joint* or orthopaedic or orthopedic or PJI or PJIs or DAIR or (prosthetic adj joint adj infection*)).ti,ab,kw.	1951367
11	6 and 9 and 10	1328
12	limit 11 to english language	1245

***Web of Science* - Total number of references found – 571**

Searched 29 May 2023

Database coverage (years) - 1997–present [Science Citation Index Expanded (Sci-Expanded), Social Science Citation Index (SSCI), Arts & Humanities Citation Index (AHCI). 2018–present [Emerging Sources Citation Index (ESCI)]:

#	Searches	Results
1	TOPIC: (periprosthe* or peri-prosthe* or arthroplast* or implant or metalware or hip or knee or shoulder or elbow)	704,570
2	TOPIC: ((infect* NEAR/8 (joint* or prosthe* or implant or metalware)) or PJI)	24,481
3	TOPIC: (rct or randomized or P3 or "phase III")	1,032,455
4	#3 AND #2 AND #1	759
5	#3 AND #2 AND #1 Refined by: [excluding] WEB OF SCIENCE CATEGORIES: (RADIOLOGY NUCLEAR MEDICINE MEDICAL IMAGING OR DENTISTRY ORAL SURGERY MEDICINE OR UROLOGY NEPHROLOGY OR ENDOCRINOLOGY METABOLISM OR PUBLIC ENVIRONMENTAL OCCUPATIONAL HEALTH OR OBSTETRICS GYNECOLOGY OR ZOOLOGY OR OTORHINOLARYNGOLOGY OR ANDROLOGY OR CLINICAL NEUROLOGY OR VETERINARY SCIENCES OR OPHTHALMOLOGY OR CARDIAC CARDIOVASCULAR SYSTEMS OR DERMATOLOGY OR RESPIRATORY SYSTEM OR GASTROENTEROLOGY HEPATOLOGY OR NEUROSCIENCES OR ONCOLOGY OR PARASITOLOGY)	571

Cochrane Library (including the Trials Register) - Total number of references found - 1235

Searched 7 June 2023

#	Searches	Results
1	MeSH descriptor: [Prosthesis-Related Infections] explode all trees	255
2	(arthroplasty OR orthopedic implant OR prosthetic joint or metalware):ti,ab,kw (Word variations have been searched)	15,163
3	(infection OR infected OR infections):ti,ab,kw (Word variations have been searched)	144,732
4	#2 AND #3	1481
5	#1 OR #4	1642
6	(RCT* OR random*):ti,ab,kw or randomized controlled trial	1,319,464
7	#5 AND #6	1344
8	hip or knee or knees or shoulder or elbow or joint or joints or pji or orthopaedic	119,767
9	#7 AND #8	1246

20 Cochrane Reviews
917 Journal articles
214 ClinicalTrials.gov
94 ICTYRP

[*Australian and New Zealand Clinical Trials Registry*]

Date of search - 31 May 2023

Total number of references found – 36

search terms - prosthesis or prosthetic or prostheses or arthroplasty or hip or knee

condition category - infection

WHO International Clinical Trials Registry Platform

Date of search – 31 May 2023

Total number of references found - 39

(Periprosthetic OR peri-prosthetic OR PJI) "in title"

AND Infection "in condition"

ClinicalTrials.gov

Date of search – 31 May 2023

Total number of references found – 107 (Phase 3 and Phase 4, Interventional studies only)

Study List: - **((Periprosthetic OR peri-prosthetic OR joint OR arthroplasty OR PJI) AND infection) NOT (ankylosing OR hepatitis)**

EU Clinical Trials Register

Date of search – 31 May 2023

Total number of references found – 75 (Phase 3 and Phase 4 only)

((infection AND (prosthesis OR prosthetic OR prostheses OR arthroplasty OR hip OR knee)) OR PJI) NOT HIV

Removal of duplicate records in EndNote

Clinical trials (Cochrane, WHO (ICTRP), ANZCTR, ClinicalTrials.gov, EUCTR)

The clinical trials were deduplicated manually by the Librarian (BA); some trials may be the same ones registered in different Registries, but these were left in the EndNote file. Of the 561 trials, 33 were removed as duplicates.

Journal articles (Medline, Embase, Web of Science, Pubmed, Cochrane)

EndNote deduplication protocols removed a total of 1331 duplicate records from the EndNote library containing the raw data.

Key to symbols and search terms

* = wildcard in keyword, focus on indexed term

/ = indexed term term

exp = explodes the term entered and retrieves records that contain the term and any of its narrower, more specific terms.

Based on: Moher D, Liberati A, Tetzlaff J, Altman DG, The PRISMA Group (2009). *P*referred *R*eporting *I*tems for *S*ystematic Reviews and *M*eta-*A*nalyses: The PRISMA Statement. PLoS Med 6(6): e1000097. doi:10.1371/journal.pmed1000097 [40]

PRISMA 2009 Flow Diagram

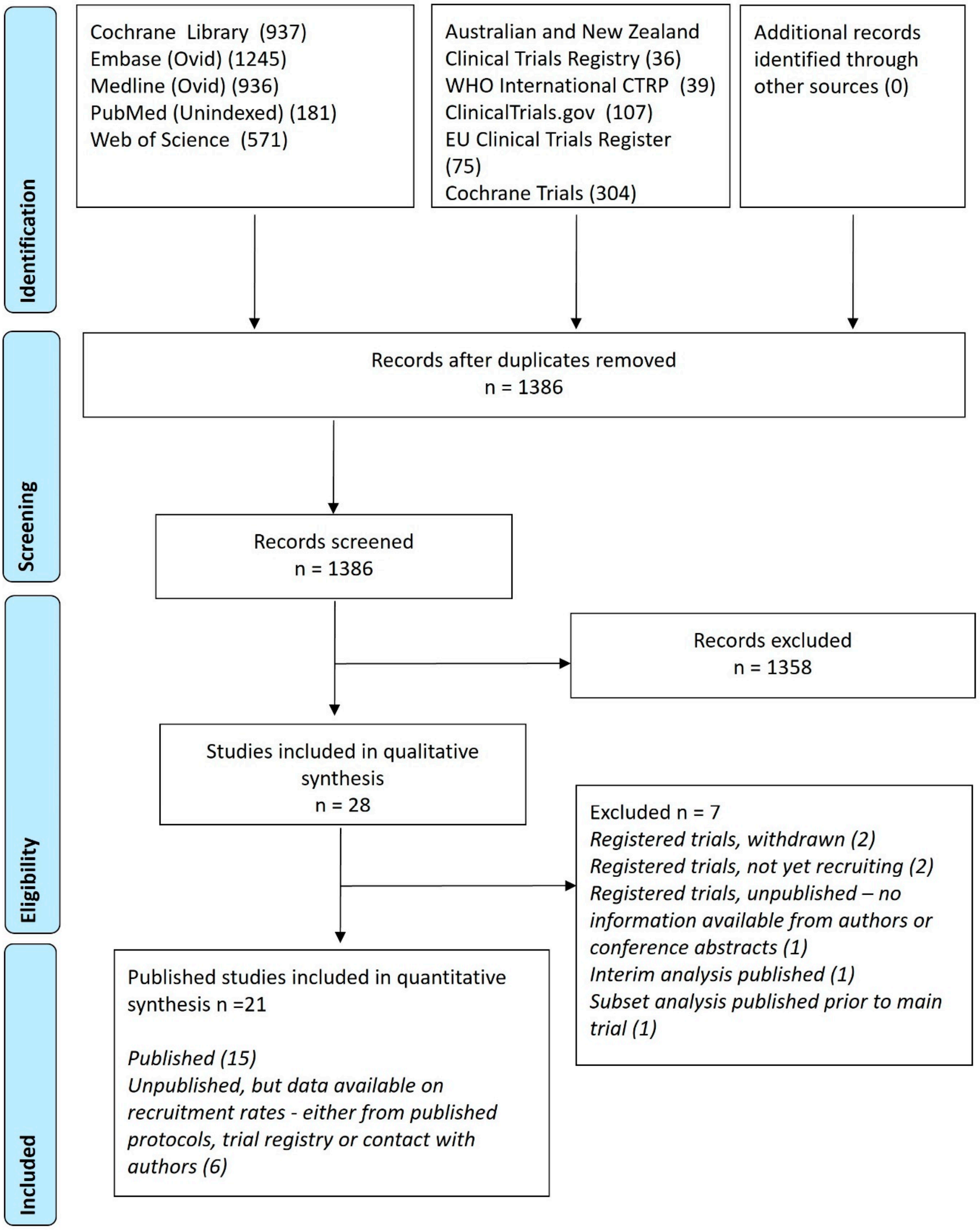

Supplement: Supplementary file 1 [file antibiotics-12-01486-s001.zip › antibiotics-2587419-supplementary.pdf]
